# Recovery of Ellagic Acid from Pomegranate Peels with the Aid of Ultrasound-Assisted Alkaline Hydrolysis

**DOI:** 10.3390/molecules29112424

**Published:** 2024-05-21

**Authors:** Anastasia Kyriakoudi, Evmorfia Kalfa, Eleni Zymvrakaki, Natasa Kalogiouri, Ioannis Mourtzinos

**Affiliations:** 1 Laboratory of Food Chemistry and Biochemistry, School of Agriculture, Aristotle University of Thessaloniki (AUTH), 54124 Thessaloniki, Greece; morfok23@gmail.com (E.K.); elenzym@agro.auth.gr (E.Z.); mourtzinos@agro.auth.gr (I.M.); 2 Laboratory of Analytical Chemistry, Department of Chemistry, Aristotle University of Thessaloniki, 54124 Thessaloniki, Greece; kalogiourin@chem.auth.gr

**Keywords:** pomegranate peels, ellagic acid, ultrasound-assisted alkaline hydrolysis, LC-QTOF-MS analysis, GC-MS analysis

## Abstract

The pomegranate processing industry generates worldwide enormous amounts of by-products, such as pomegranate peels (PPs), which constitute a rich source of phenolic compounds. In this view, PPs could be exploited as a sustainable source of ellagic acid, which is a compound that possesses various biological actions. The present study aimed at the liberation of ellagic acid from its bound forms via ultrasound-assisted alkaline hydrolysis, which was optimized using response surface methodology. The effects of duration of sonication, solvent:solid ratio, and NaOH concentration on total phenol content (TPC), antioxidant activity, and punicalagin and ellagic acid content were investigated. Using the optimum hydrolysis conditions (i.e., 32 min, 1:48 *v*/*w*, 1.5 mol/L NaOH), the experimental responses were found to be TCP: 4230 ± 190 mg GAE/100 g dry PPs; A_ABTS_: 32,398 ± 1817 µmol Trolox/100 g dry PPs; A_CUPRAC_: 29,816 ± 1955 µmol Trolox/100 g dry PPs; 59 ± 3 mg punicalagin/100 g dry PPs; and 1457 ± 71 mg ellagic acid/100 g dry PPs. LC-QTOF-MS and GC-MS analysis of the obtained PP extract revealed the presence of various phenolic compounds (e.g., ellagic acid), organic acids (e.g., citric acid), sugars (e.g., fructose) and amino acids (e.g., glycine). The proposed methodology could be of use for food, pharmaceutical, and cosmetics applications, thus reinforcing local economies.

## 1. Introduction

Pomegranates (*Punica granatum* L.) are usually consumed either fresh or in the form of juices, jams, jellies, liqueurs, etc. Considering that the global production of pomegranates was estimated to be ~3.5 million tonnes in 2017 [1], the pomegranate processing industry generates annually enormous amounts of by-products and wastes that are mainly composed of seeds and peels. The latter ones account for about 40% of the whole fruit weight, and their disposal constitutes a major issue for the pomegranate processing industry worldwide. Till today, pomegranate peels (PPs) are either discarded in landfill sites, used as animal feed [2], or used for energy production (e.g., bioethanol) [3,4,5].

Pomegranate peels, apart from carbohydrates, crude fibers, crude protein, and aromatic amino acids, contain high amounts of secondary metabolites, including flavonols, anthocyanins, phenolic acids, gallotannins, and ellagitannins [6]. PPs have been reported to contain higher amounts of phenolic compounds compared to other agroindustrial by-products (e.g., apple peels, black carrot peels, grape pomace, etc.). Even though the majority of phenolic compounds of PPs are found in free soluble form, significant amounts are also found in bound forms [7]. In particular, ellagitannins, often in the form of hexahydroxydiphenic acid (HHDP)-galloyl-hexoside, i.e., punicalagins, are one of the major phenolic compounds present in PPs. Upon alkaline, acidic, or enzymatic hydrolysis, ellagitannins release the HHDP group, which after spontaneous lactonization results in the formation of ellagic acid [8]. The latter one is a dimeric gallic acid derivative of high commercial value considering that it has been reported to possess various biological actions such as antioxidant, anti-inflammatory, antiatherosclerotic, antiviral, and antiobesity, as well as cardio-, gastro-, and neuroprotective properties [9]. It has been reported that ellagic acid can be also released from ellagitannins after their hydrolysis in the small intestine. Even if free ellagic acid is considered to be poorly absorbed, it can be converted by gut microbiota to bioavailable metabolites, namely urolithins, that exhibit chemopreventive, antiatherosclerotic, and anti-inflammatory action [10]. Apart from PPs, which constitute one of their richest source, ellagic acid is also found in strawberries, raspberries, blackberries, and other types of berries, as well as certain nuts (e.g., walnuts, pistachio, cashew) [11].

Until now, the focus has been on extracting free phenolic compounds from PPs using a variety of solvents, such as water, ethanol, and methanol, as well as their mixtures (e.g., [12,13]). However, to the best of our knowledge, extremely limited are the data regarding the recovery of free and bound phenolic compounds, including ellagic acid from PPs [14]. Until now, hydrolysis has been performed using time-consuming conventional techniques that require substantial amounts of solvents and have a low recovery yield. For example, this is illustrated in the work of Liu et al. [15], who treated PPs with 0.5 mol/L sodium hydroxide (NaOH) for 3 h upon stirring. In the same frame, Dadwal et al. [16] performed alkaline hydrolysis for the extraction of bound phenolics from PPs using 4 mol/L NaOH for 4 h with the aid of continuous shaking at room temperature. Sun et al. [17] carried out acidic hydrolysis of PPs with 6 mol/L hydrochloric acid (HCl) for 2 h using a water bath at 45 °C. Towards bioethanol production, Chaudhary et al. [18] carried out alkaline hydrolysis of PPs using 0.5% KOH at 80 °C for 90 min, and Saleem et al. [19] employed acidic hydrolysis of PPs with 5% acid concentration at 100 °C for 30 min, without mentioning the means of extractions. Ultrasound-assisted extraction was employed for the recovery of ellagic acid by Μuniz-Marquez et al. [8]; however, these authors used ethanol as the extraction solvent and concluded that the obtained yield was low, since the release of ellagic acid was attributed only to cell rupture due to the effect of ultrasounds and not in the hydrolysis of ellagitannins. Moreover, hydrolysis of the ellagitannins of pomegranate wastes towards the recovery of ellagic acid has been also carried out using solid state fermentation with *Saccharomyces cerevisiae* and *Aspergillus niger* [20].

In this context, the objective of the current work was to systematically investigate the effect of ultrasound-assisted alkaline hydrolysis conditions (i.e., duration, solvent:solid ratio, NaOH concentration) on the total phenol content (TPC), the antioxidant activity (DPPH^●^, ABTS^●+^, CUPRAC assays), and on the recovery of the major phenolic compounds, i.e., punicalagin and ellagic acid, of the obtained extracts derived from PPs originating from a Greek pomegranate processing industry. The ultrasound-assisted alkaline hydrolysis conditions were optimized using response surface methodology (RSM). The degree of hydrolysis of punicalagin and the concomitant liberation of ellagic acid was monitored by HPLC-DAD. Moreover, the PP extract prepared under the optimum hydrolysis conditions was further characterized with the aid of liquid chromatography–quadrupole time-of-flight mass spectrometry (LC-QTOF/MS) and gas chromatography–mass spectrometry (GC-MS). The proposed methodology could pave the way for PP exploitation as a sustainable source of ellagic acid, as well as of other valuable phenolic compounds that could be used for novel food, pharmaceutical, and cosmeceutical applications.

## 2. Results and Discussion

### 2.1. Extraction of Free Phenolics from Pomegranate Peels

Initially, the free phenolic compounds of PPs were extracted with a mixture of ethanol:water 50:50 (*v*/*v*) that was found to be the most effective solvent system in our recent study [12]. The TPC, A_DPPH_, A_ABTS_, and A_CUPRAC_, as well as punicalagin (sum of α and β anomeric forms) and ellagic acid content in the free fraction were found to be 10,159 ± 395 mg GAE/100 g dry PPs, 39,205 ± 237 µmol Trolox/100 g dry PPs, 1593 ± 139 µmol Trolox/100 g dry PPs, 84,419 ± 1069 µmol Trolox/100 g dry PPs, 1197 ± 79 mg punicalagin/100 g dry PPs, and 515 ± 19 mg ellagic acid/100 g dry PPs, respectively. The punicalagin content found in the present study is similar to that reported by Gullon et al. [21] (i.e., 1667 mg/100 dry weight), who extracted phenolics from PPs with the aid of ultrasounds using 50% aqueous ethanol as well. Kaderides et al. [13] employed microwave-assisted extraction and reported a punicalagin content of 14,364 mg/100 g dry weight. Even higher values (i.e., 24,547 mg/100 g dry weight) have been reported by Kharchoufi et al. [22], who used water as the extraction solvent upon stirring. Regarding the ellagic acid content found in the present study, it was found to be almost half of that of punicalagin. An ellagic acid content of 39–304 mg/100 g dry weight has been reported by Yan et al. [23], who carried out the extraction using a 80% methanol–water solution with the aid of a Soxhlet apparatus at 80 °C. Sabraoui et al. [24] examined different varieties of pomegranates grown in Morocco and reported an ellagic acid content of 160–3500 mg/100 g dry weight after extracting PPs with methanol using magnetic stirring at room temperature for 24 h. Such quantitative variations could be associated with the different extraction means and solvents, as well as with differences in maturity stage and the geographical origin of the fruits [22].

### 2.2. Extraction of Bound Phenolics from Pomegranate Peels

#### 2.2.1. Model Fitting for TPC, A_DPPH_, A_ABTS_, A_CUPRAC_, Punicalagin and Ellagic Acid Content

The dried solid residue that remained after the extraction of free phenolics was then used for the liberation of bound phenolic compounds from PPs with the aid of ultrasound-assisted alkaline hydrolysis. RSM was applied to investigate the effects of the duration of ultrasound-assisted alkaline hydrolysis (X_1_), solvent:solid ratio (X_2_), and NaOH concentration (X_3_) on the TPC, A_DPPH_, A_ABTS_, and A_CUPRAC_, as well as on punicalagin and ellagic acid content. In the present study, ultrasounds were selected as the means of extraction in order to avoid high temperatures that occur during microwave-assisted extraction, as has been reported by Kaderides et al. [25]. The experimental responses (Table 1) for all the examined variables were analysed by ANOVA (Table 2) in order to test the validity of each model. The experimental data in every case were fitted to the second-order polynomial model. The DPPH^●^ scavenging activity model showed a nonsignificant (*p* < 0.05) lack of fit. On the other hand, the models for TPC, A_ABTS_, and A_CUPRAC_, as well as punicalagin and ellagic acid content, displayed a statistically significant regression (*p* < 0.05) and R^2^ values ranging from 0.858 to 0.996, thus indicating that they could explain >85% of the variability of the responses. The second-order polynomial equations (Equations (1)–(5)) obtained for the five responses (Models A–E) are shown in Table 3.

#### 2.2.2. Major Effects of Ultrasound-Assisted Alkaline Hydrolysis Conditions

##### Effects on Total Phenol Content

**Figure 1:**
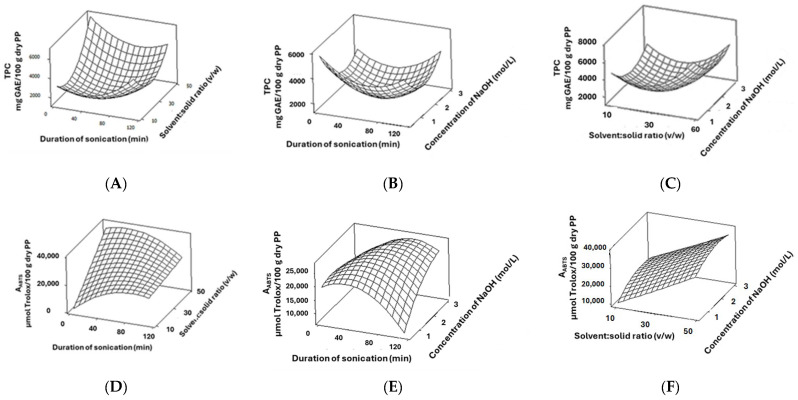

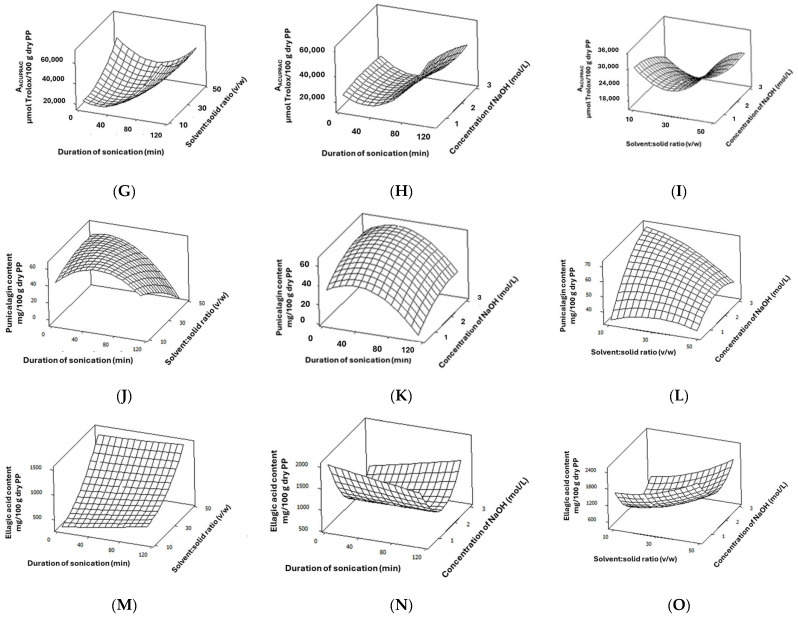
Surface plots for total phenol content (**A**–**C**), ABTS^●+^ scavenging activity (**D**–**F**), and cupric ion reducing antioxidant capacity (**G**–**I**), as well as punicalagin (**J**–**L**) and ellagic acid content (**M**–**O**) values affected by duration of sonication, solid:solvent ratio, and NaOH concentration. In all cases, the third factor was kept constant at its middle level.

The TPC of the obtained PP extracts was found to range from 1364 to 5976 ± 173 mg GAE/100 g dry PPs (Table 1). The analysis of variance for the TPC values (Model A) showed that both linear and quadratic X_2_, as well as the quadratic X_1_ and X_3_, exhibited a significant and positive effect, whereas the respective linear terms were statistically nonsignificant. Figure 1A–C show the generated three-dimensional surface plots for each pair of factors, thereby keeping the third one constant at its middle level (Table 1). As it is illustrated, the TPC reached its highest absolute values for short sonication duration at a high solvent:solid ratio and at the middle levels of NaOH concentration.

##### Effects on In Vitro Antioxidant Activity

The antioxidant activity values of the PP extracts using the DPPH^●^ assay varied from 8810 to 69,832 ± 390 μmol Trolox/100 g dry PPs. A statistically insignificant regression model was found for DPPH^●^ scavenging activity. The lack of a statistically significant model for DPPH^●^ has been reported also by Kyriakoudi et al. [26], who optimized the microwave-assisted alkaline hydrolysis of rice hulls, as well as by Pyrka et al. [27], who optimized olive leaves’ thin layer using intermittent near-infrared drying. These authors attributed this finding to the synergistic and antagonistic phenomena of phenols in extracts (Olszowy-Tomczyk, 2020) [28] that minimize differences in the estimated antioxidant activity, despite variances in their phenolic content. On the contrary, the model for ABTS^●+^ scavenging activity described 85.77% of the variability of the responses (Table 3). The linear X_2_ and quadratic X_1_ were found to have significant effects. As it is shown in the respective surface plots in Figure 1D–F, a maximum ABTS^●+^ activity could be obtained using a high solvent:solid ratio and low sonication duration when keeping the NaOH concentration at its middle level. A statistically significant model was also found for the CUPRAC assay, which is also based on an electron transfer mechanism, like Folin–Ciocalteau, thus indicating that ultrasound-assisted alkaline hydrolysis affects the redox potential of PP extracts. A similar correlation between the results obtained with Folin–Ciocalteau and CUPRAC assays has been also observed by Pyrka et al. [27]; these authors suggested that drying affects the redox status of olive leaves. The duration of ultrasound-assisted alkaline hydrolysis (X_1_) showed significant linear and quadratic effects on the CUPRAC response. In Figure 1G–I, a trend toward higher A_CUPRAC_ values can be noted upon increasing the solvent:solid ratio up to high levels.

##### Effects on Punicalagin and Ellagic Acid Content

A statistically significant regression model was also found for punicalagin. The model for punicalagin could describe 80.50% of the variability of the responses (Table 3). The linear terms of duration of the sonication, solid:solvent ratio and NaOH concentration (X_1_, X_2_, and X_3_), as well as the quadratic terms of the duration of sonication and NaOH concentration, were found to have a significant effect on the punicalagin content. According to the surface plots shown in Figure 1J–L, the punicalagin content values were found to decrease upon increased duration of sonication and low NaOH concentration, which was probably due to its hydrolysis. Moreover, the model for ellagic acid content could describe 99.58% of the variability of the responses (Table 3), with the linear and quadratic effects of X_1_ having a significant negative and positive effect, respectively. Moreover, the linear and quadratic effects of X_2_, as well as the quadratic effect of X_3_, had a significant positive effect, whereas the X_1_–X_2_ interaction had a significant negative effect. As shown in the corresponding surface plots in Figure 1M–O, the maximum ellagic acid content values could be obtained when a short duration of sonication and a low NaOH concentration were used.

**Figure 2:**
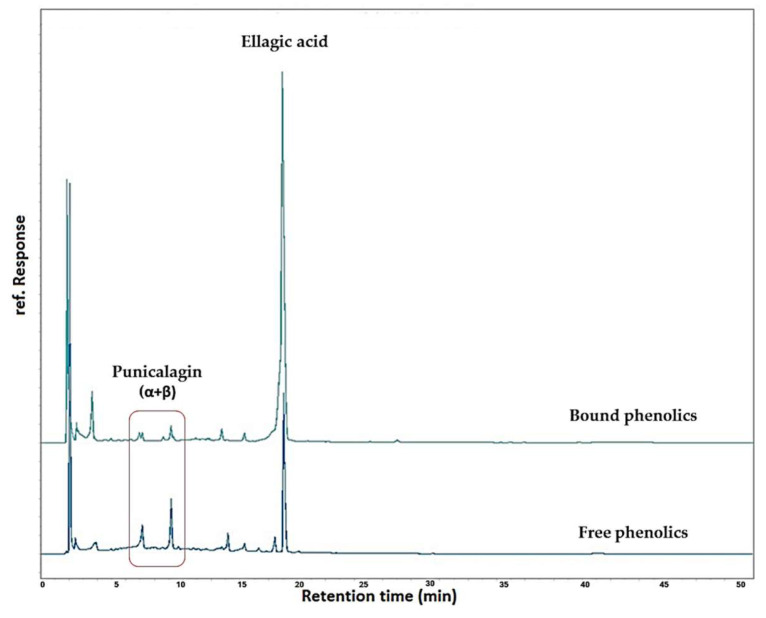
RP-HPLC-DAD chromatograms at 280 nm of PP extracts before (free phenolics) and after (bound phenolics) ultrasound-assisted alkaline hydrolysis.

As can be seen in Table 2, the levels of punicalagin (sum of α and β anomeric forms) were found to range from 22 to 109 mg/100 dry PP. As can be observed, these values are much lower than that reported for the punicalagin content in the free fraction prior to alkaline hydrolysis. On the contrary, the ellagic acid content was found to increase after hydrolysis. In particular, the ellagic acid content after hydrolysis was found to range from 727 to 1828 mg/100 g dry PPs compared to the 515 ± 19 mg/100 g dry PPs that was in the free fraction. A RP-HPLC-DAD chromatogram showing the punicalagin and ellagic acid before and after ultrasound-assisted alkaline hydrolysis is given in Figure 2.

#### 2.2.3. Multiple Response Optimisation for Ultrasound-Assisted Alkaline Hydrolysis Conditions

The independent variables, namely the duration of ultrasound-assisted alkaline hydrolysis, the solvent:solid ratio, and the NaOH concentration, that were determined to be optimum for the responses TPC, A_ABTS_, and A_CUPRAC_, as well as punicalagin and ellagic acid content, using the RSM multiple response optimization approach are shown in Table 4. As can be seen, the predicted values fit well with the experimental ones of the respective responses (the mean % difference between the predicted and the experimental values was 8.6%, which is considered satisfactory).

### 2.3. Target and Nontarget LC-QTOF-MS Analysis

The targeted LC-QTOF-MS analysis revealed the presence of a variety of bound phenolic compounds present in the examined PP extract prepared under the optimum hydrolysis conditions. More specifically, as it is shown in Table 5, out of the twenty examined target compounds, eight phenolic compounds were determined in the examined PP extract. In particular, from the initial target list, the flavonoids apigenin, catechin, kaempferol, luteolin, quercetin, rutin, and taxifolin, as well as the phenolic acid, namely protocatechuic acid, were identified and quantified based on appropriate standard calibration curves, (i.e., apigenin: y = 2 × 10^7^x + 8 × 10^6^, catechin: y = 9 × 10^6^x + 994,352, kaempferol: y = 2 × 10^7^x + 5 × 10^6^, luteolin: y = 3 × 10^7^x + 7 × 10^6^, protocatechuic acid: y = 2 × 10^6^x−31,489, quercetin: y = 2 × 10^7^x + 2 × 10^6^, rutin: y = 9 × 10^6^x − 703,039, and taxifolin: y = 8 × 10^6^x + 706,635) that were linear over the range 0.01–5 mg/L (R^2^ > 0.999). The concentrations of these compounds in the examined PP extract were found to range from 0.05 µg/mL for quercetin and taxifolin to 1.04 µg/mL for protocatechuic acid.

Apart from the above-mentioned phenolic compounds, the nontarget analysis of the prepared PP extract revealed the present of other phenolic compounds belonging to the classes of hydrolysable tannins and phenolic acids. In particular, ellagic acid, that was liberated during the ultrasound-assisted alkaline hydrolysis from ellagitannins such as punicalagin, was tentatively identified in the samples using the SCIEX Natural Products Library and Formula Finder algorithm, with a score above 50.0. Similarly, caffeic acid, ferulic acid, and p-coumaric acid were also tentatively identified. The chromatograms and MS/MS fragmentation patterns of the target compounds are given in Table S1 of the Supplementary Materials. Many compounds have been identified in PP extracts from various researchers. For example, Ambigaipalan et al. [29] investigated the phenolic compounds of pomegranate by-products of fruits grown in California, and they identified gallic acid as the major phenolic acid in addition to p-coumaric acid, ellagic acid, kaempferol 3-O-glucoside, and procyanidin dimers in insoluble-bound phenolic samples that were subjected to alkaline hydrolysis by UPLC-DAD-ESI-MS. Dadwal et al. [16] identified and quantified punicalagin and catechin-bound phenolics in fresh PPs. Moreover, gallic acid, catechin, ellagic acid, and rutin, along with luteolin-7-O-glucoside, punicalagin, quercetrin-3-O-glucoside, and apigenin-7-glucoside, have been identified in PPs derived from cultivars from China with the aid of UHPLC-QTOF-MS and UPLC-QQQ-MS [30]. The presence of ellagic acid, p-coumaric acid, and catechin has been also confirmed in PP extracts that were prepared using thermal and nonthermal extraction methods by Man et al. [14] using UHPLC-QTOF-MS. Apart from these compounds, the authors also identified gallic acid, epicatechin, gallocatechin, epigallocatechin, and punicalin, as well as α-punicalagin and β-punicalagin.

### 2.4. GC-MS Analysis

**Figure 3:**
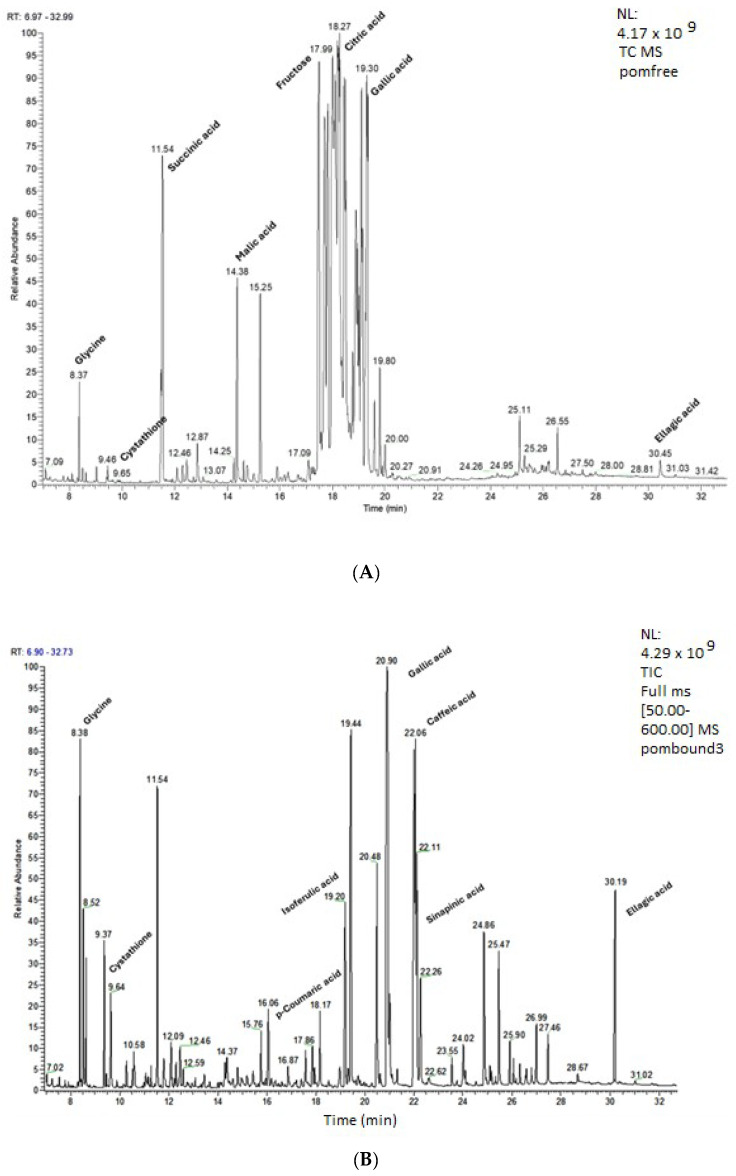
GC-MS profile of PP extracts prepared without (**A**) and with (**B**) ultrasound-assisted alkaline hydrolysis.

GC coupled to a mass detector was also employed to obtain further evidence for the identity of compounds present in the PP extract prepared under the optimum ultrasound-assisted alkaline hydrolysis. For comparison purposes, the profile of the PP extract prepared without hydrolysis was also examined. The extracts were analysed after silylation. The latter one is a procedure that allows the GC analysis of nonvolatile and thermolabile compounds. In the present study, BSTFA has been used for the preparation of trimethylsilyl (TMS) derivatives, as has been previously reported for other plant materials (e.g., [31,32]). The GC-MS analysis revealed a different profile between the two examined PP extracts (Figure 3A,B). As can be observed, the PP extract prepared without hydrolysis exhibited a more complex profile compared to that of the extract after the alkaline hydrolysis. As can be seen in Table 6 and Figure 3A, various sugars and sugar alcohols, namely D-mannitol, fructose, D-sorbitol, D-glycose, and myoinositol, were identified. Moreover, the GC-MS analysis revealed the presence of certain amino acids, namely glycine and cystathione, as well as organic acids such as succinic acid, malic acid, and citric acid. Additionally, phenolic acids, such as gallic acid, caffeic acid, p-coumaric acid, and ellagic acid, were also identified, thus verifying also the results of the nontarget LC-QTOF-MS analysis. It is worth mentioning that citric acid and gallic acid were the most abundant constituents of the examined PP extract. Punicalagin, also present in high amounts in the examined PP extract as evidenced by the HPLC-DAD analysis, was not identified by GC-MS, because its molecular weight was above the mass range examined in the present study. Even though, to the best of our knowledge, no data exist regarding the analysis of PP extracts using GC-MS, all the above-mentioned compounds are expected to be present in PPs [33].

On the other hand, ultrasound-assisted alkaline hydrolysis was found to cause remarkable changes in the profile of the PP extract. As depicted in Figure 3B, the hydrolyzed PP extract exhibited a diverse profile showcasing the presence of various compounds, such as phenolic acids. Among the identified compounds, gallic acid, along with ellagic acid that was liberated from its bound forms upon hydrolysis as verified by the HPLC-DAD analysis, were some of the major compounds identified in the examined extract. Even though ellagic acid was not the predominant compound in the hydrolyzed PP extract based on the GC-MS analysis, we chose to focus on it, thus considering that PPs constitute one of ellagic acid’s main sources. Moreover, the GC-MS analysis revealed a substantial increase in ellagic acid abundance following its liberation from bound forms during hydrolysis.

## 3. Materials and Methods

### 3.1. Reagents and Solvents

Ellagic acid (≥95%) was purchased from Extrasynthese (Genay, Cedex, France). Punicalagin (≥98%) was from Glentham Life Sciences (Corsham, UK). The 6-Hydroxy-2,5,7,8-tetramethyl-chroman-2 acid (Trolox) (97%), 3,4,5-trihydroxybenzoic acid (gallic acid) (99%), apigenin (98%), catechin (98%), chlorogenic acid (98%), epicatechin (98%), isorhamnetin (98%), kaempferol (98%), luteolin (98%), protocatechuic acid (98%), quercetin (98%), quercitrin (98%), rutin (98%), and taxifolin (98%) were all purchased from Sigma-Aldrich (Stenheim, Germany). Ammonium acetate (CH_3_COONH_4_) (99%), hydrochloric acid (HCl, 37% *w*/*w*), Folin–Ciocalteu reagent, sodium carbonate (Na_2_CO_3_, 99.8%), sodium sulphate (Na_2_SO_4_) (99%), potassium chloride (KCl) (99.5%), sodium chloride (NaCl) (99.8%), sodium dihydrochloride monoacid phosphate (Na_2_HPO_4_·2H_2_O) (99.5%), potassium dihydrgen phosphate (KH_2_PO_4_) (99.5%), sodium hydroxide (NaOH) (99%), and BSFTA [N,O-bis(trimethylsilyl)trifluoroacetamide] were from Chem-Lab (Zedelgen, Belgium). Pyridine (anhydrous 99.5%) was from Thermo Scientific (Kandel, Germany). The radical 2,2-diphenyl-1-picrylhydrazyl (DPPH^●^) (>97%) was from TCI (Kita-Ku, Tokyo, Japan). Copper dichloride dihydrate (CuCl_2_·2H_2_O) (99.99%) was from ThermoFisher (Kandel, Germany), whereas neocuproine (2,9-dimethyl-1, 10-phenanthroline) (≥98%) and the bis-ammonium salt of 2,2′-azino-bis(3-ethylbenzothiazolin-6-sulfonic acid) (ABTS) (≥98%) were from Sigma-Aldrich GmbH (Buchs, Switzerland). Potassium persulfate (K_2_S_2_O_8_) was from Merck (Darmstadt, Germany). Glacial acetic acid, as well as HPLC grade acetonitrile (>99.9%), methanol (>99.8%), and ethyl acetate (>99.8%), were from Chem-Lab (Zedelgen, Belgium). Ultrahigh-purity water was produced in the laboratory using a Micromatic Wasserlab system (Wasserlab, Barañain, Spain). LC-MS grade methanol, water, and formic acid (98–100%) were from Merck (Darmstadt, Germany).

### 3.2. Plant Material

The pomegranate fruits used in the present study belonged to the “Wonderful” variety, and they were supplied by local producers (Rodi Hellas, Greece). PPs were manually separated. The peels were freeze-dried (HyperCOOL HC8080 freeze dryer, Gyrozen Co., Ltd., Gimpo, Republic of Korea) (−80 °C, 0.1 mbar) and homogenized in a Pulverisette 11 Knife Mill (Fritsch GmbH, Idar-Oberstein, Germany) at 6000 rpm for 20 s, and they were sieved using a sieve shaker (Retax, Labor Siebmaschine, type LS10, No. 4082, Hemmingen, Germany). The average particle size was < 0.22 mm. The pomegranate peel powder was defatted with the aid of a Soxhlet extraction using diethyl ether as solvent in order to ensure the stability of the sample during storage and handling [16].

### 3.3. Extraction of Free Phenolics Compounds from Pomegranate Peels

Initially, free phenolic compounds were extracted from PPs using a mixture of water:ethanol, 50:50 (*v*/*v*) that was found to be the most efficient extraction system based on the results of our previous study [12]. In particular, extraction was carried out with the aid of a sonication bath (Elmasonic S 60 H, Elma, Singen, Germany). The duration of the extraction was 10 min. Sample temperature was kept at ~30 °C using an ice bath. Afterwards, the mixture was centrifuged (6000 rpm, 15 min), and the supernatant was collected and filtered using 0.45 μm PTFE filters and stored in the fridge (4 °C) until analysis for the determination of the free phenolic compounds of PP [12]. The remaining solid residue was then subjected to ultrasound-assisted alkaline hydrolysis in order to liberate bound phenolic compounds from PPs.

### 3.4. Experimental Design for Optimising the Ultrasound-Assisted Alkaline Hydrolysis of Pomegranate Peels towards the Recovery of Ellagic Acid

Different amounts of dried solid residues of PP after extraction of the free phenolic compounds as described above were weighted directly into Duran bottles with screw caps, and 20 mL of appropriate concentrations of NaOH in the range of 0.5–4 mol/L were added and subjected to ultrasound-assisted alkaline hydrolysis. At the end of each process, the mixture was centrifuged at 6000 rpm for 15 min. The supernatant was collected, and the pH value was adjusted to 1 with 12 mol/L HCl in an ice bath. In order to remove precipitated material, the solution was centrifuged once again (6000 rpm, 15 min). The collected supernatant was then extracted with ethyl acetate (3 × 50 mL). The ethyl acetate fractions were collected, combined, and dried using anhydrous sodium sulfate. Ethyl acetate was then evaporated to dryness, and the dry residue was redissolved in 10 mL methanol for subsequent studies.

Experiments aiming to optimize the ultrasonic-assisted alkaline hydrolysis of PPs were designed with the aid of Minitab 15.1.20.0 (Minitab, Inc., State College, PA, USA) software using an unblocked full factorial central composite design (CCD) of the response surface methodology. Each one of the three independent variables, namely duration of hydrolysis (min) (X_1_), solvent:solid ratio (X_2_), and NaOH concentration (X_3_), had five experimental levels coded as −a, −1, 0, +1, +a (a = 1.41421), where −1, +1 and 0 correspond to the low, high, and middle levels (Table 7). A total of 20 experimental runs with six center points were conducted according to the experimental design (Table 1). The responses that were examined included the total phenol content (TPC) (Y_1_), DPPH^●^ (A_DPPH_) (Y_2_) and ABTS^●+^ (A_ABTS_) (Y_3_) radical scavenging activity, cupric ion reducing antioxidant capacity (A_CUPRAC_) (Y_4_), and the punicalagin (Y_5_) and ellagic acid content (Y_6_). Each experimental response was analysed, and a second-order regression equation (Equation (6)) was obtained:

Y = β_0_ + β_1_X_1_ + β_2_X_2_ + β_3_X_3_ + β_11_X_12_ + β_22_X_22_ + β_33_X_32_ + β_12_X_1_X_2_ + β_13_X_1_X_3_ + β_23_X_2_X_3_ (6)

where Y corresponds to the responses, X_1_, X_2_, and X_3_ represent the factors of duration, temperature, and solvent:solid ratio, and β_0_, β_1_, .... β_23_ are the estimated coefficients, with β_0_ being a scaling constant.

Analysis of variance (ANOVA) was carried out to evaluate the quality of the fit of the model to the responses by investigating the coefficients of determination (R^2^), the significance of each parameter through the F-test (*p*-value), and the lack of fit of the model. Coefficients with a *p* value lower than 0.05 were considered statistically significant. Multiresponse optimisation of the fitted polynomials was also performed using the Minitab software.

### 3.5. Spectrophotometric Determinations

#### 3.5.1. Determination of the Total Phenol Content (TPC)

Total polar phenol content of the prepared PP extracts was determined spectrophotometrically with the Folin–Ciocalteu assay according to the procedure described by Kyriakoudi et al. [26]. Gallic acid was used as a reference standard, and results were expressed as gallic acid equivalents (mg GAE/100 g dry PP). In a 10 mL volumetric flask, 5 mL of water with the appropriate amount of pomegranate peel extract and 0.5 mL Folin–Ciocalteu reagent were added. After 3 min, 1.0 mL of saturated sodium carbonate solution (37%, *w*/*v*) was added, and the mixture was agitated. The volume was adjusted with water, and the flask left in the dark for 1 h at room temperature. The absorbance was measured at 750 nm (UV-1800 spectrophotometer, Shimadzu, Kyoto, Japan) against a blank prepared similarly using methanol instead of the extract. Measurements were performed at least in triplicate, and results were expressed as the mean value ± s.d.

#### 3.5.2. DPPH^●^ Scavenging Activity (A_DPPH_)

The DPPH^●^ scavenging activity of PP extracts was determined according to the procedure described by Kyriakoudi et al. [26]. Appropriate amounts of pomegranate peel extracts were added to 2.9 mL of a 0.1 mM methanolic solution of DPPH^●^. The absorbance at 515 nm was recorded at the start and after 30 min using the UV-1800 spectrophotometer. Radical scavenging activity (%) values (%RSA) were determined by using the formula %RSA = [Abs515(t = 0) − Abs515(t)] × 100/Abs515(t = 0) after correction with appropriate blank. These values were applied to a calibration curve constructed using Trolox as a reference compound, and the results were finally expressed as μmol Trolox/100 g dry PPs. Measurements were performed at least in triplicate, and results were expressed as the mean value ± s.d.

#### 3.5.3. ABTS^●+^ Scavenging Activity (A_ABTS_)

Radical scavenging activity of PP extracts against ABTS^●+^ was evaluated according to the protocol of Re et al. [34]. In particular, the ABTS^●+^ solution was prepared by reaction of 5 mL of a 7 mM aqueous ABTS solution and 88 μL of a 140 mM potassium persulfate (K_2_S_2_O_8_) solution. After storage in the dark for 16 h, the radical cation solution was further diluted in PBS (pH 7.4) until the initial absorbance value of 0.70 (±0.05) at 734 nm was reached. An aliquot of each PP extract was mixed with 2 mL of the ABTS^●+^ solution. The decrease in absorbance was recorded at 0 and after 6 min (UV-1800 spectrophotometer). Inhibition of ABTS radical cation in percent (% Inh) was calculated by using the formula % Inh = [Abs734_(t=0)_ – Abs734_(t=6)_] × 100/Abs734_(t=0)_ after correction with an appropriate blank. These values were applied to a calibration curve constructed using Trolox as a reference compound, and the results were finally expressed as μmol Trolox/100 g dry PPs. Measurements were performed at least in triplicate, and results were expressed as the mean value ± s.d.

#### 3.5.4. Cupric ion Reducing Antioxidant Capacity (A_CUPRAC_)

The Cu (II) reducing capacity of PP extracts was measured according to the protocol of Apak et al. [35]. Briefly, 1 mL of a 0.02 M solution of cooper (II) chloride, 1 mL of a 0.0075 Μ neocuproine solution, and 1 mL of a 1 M ammonium acetate buffer (pH = 7.0) were mixed with an appropriate amount of each PP extract. After the addition of deionised water to a final volume of 4.1 mL, the mixture was shaken for 15 s. The absorbance at 450 nm was measured after the solution had been allowed to stand in the dark for 30 min (UV-1800 spectrophotometer). These values were applied to a calibration curve constructed using Trolox as a reference compound, and the results were finally expressed as μmol Trolox/100 g dry PPs. Measurements were performed at least in triplicate, and results were expressed as the mean value ± s.d.

### 3.6. RP-HPLC-DAD Analysis of Phenolic Compounds

The contents of punicalagin and ellagic acid were determined by RP-HPLC-DAD. The HPLC system consisted of an Agilent 1260 Infinity II Quaternary Pump VL, an Agilent 1260 Infinity II Autosampler, and an Agilent 1260 Infinity II Diode Array Detector High Sensitivity. Separation was carried out on a InfinityLab Poroshell 120 EC-C184 μm (150 × 4.6 mm i.d.) column (Agilent Technologies, Santa Clara, CA, USA). The column temperature was set at 30 ℃. The mobile phase consisted of water–acetic acid (0.5%, *v*/*v*) (A) and acetonitrile (B). The elution protocol was based on the method described by Kaderides et al. (2019) [13]: 0–20 min for 5% (B); 20–40 min for 25% (B); 40–45 min for 50% (B); and 45–50 min for 5% (B). The total run time was 50 min with flow rate of 0.8 mL/min, and injection volume was 20 µL. Extracts were analysed after proper dilution (when required) and filtration through 0.45 μm PTFE filters (Frisenette, Knebel, Denmark). Monitoring was in the range of 190–600 nm. Chromatographic data were processed using the OpenLab CDS version 3.5 software (2021, Agilent Technologies, Santa Clara, CA, USA). Peak identification was based on retention times and spectral characteristics (absorption maxima) with those of available standards. Quantification of punicalagin (sum of α and β anomeric forms) and ellagic acid (mg/100 g dry PP) was carried out with the aid of calibration curves of properly diluted solutions of available standards: (i) punicalagin (y = 3.180x − 67.335, R^2^ = 0.999, λ_max_ = 254 nm) and (ii) ellagic acid (y = 12.485x − 46.950, R^2^ =0.999, λ_max_ = 280 nm).

### 3.7. LC-QTOF-MS Analysis

A PP extract prepared under the optimum experimental conditions was further characterised using LC-QTOF-MS. Analysis was performed using an ExionAC LC system (SCIEX, Framingham, MA, USA) that was equipped with two pumps, a solvent degasser, an autosampler, and a controller. The X500R Q-TOF mass spectrometer (SCIEX, Framingham, MA, USA) equipped with an electrospray ionization (ESI) turboVTM source was connected to the LC-system, and it was operated in the negative ionization mode. TOF-MS and TOF-MS/MS data were acquired using a data-dependent acquisition (IDA) electrospray ionisation mode. Separation was carried out using a Fortis C18 column (100 mm length, 2.1 mm i.d., 2.6 µm particle size) provided by Fortis (Cheshire, UK). The temperature of the column was 40 °C. The solvents of the mobile phase were (A) aqueous solution of 0.1% *v*/*v* formic acid and (B) methanolic solution of 0.1% *v*/*v* formic acid. The elution program was gradient, and the flow rate was set at 0.2 mL/min and 99% (A), thus gradually dropping to 61% (A) for the next 4 min. The aqueous phase dropped even further to 5% until the 12th min, and it remained stable until the 15th min at a flow rate of 0.4 mL/min. The initial conditions were restored within one min, and for the last four minutes, the aqueous phase was again at 99% at a flow rate of 0.2 mL/min to re-equilibrate the column. The QTOF-MS system was equipped with an ESI interface operating in a negative mode with the following settings: spray voltage of –4500 V, 550 °C heater gas temperature, 80 V declustering potential. The MS/MS spectra were obtained at a collision energy of 45 V and a collision energy spread of 15 V. External calibration was performed before analysis with a cluster solution provided by SCIEX, and additionally, the calibration solution was injected at the beginning of each run for internal calibration and once per five samples during batch acquisition. Mass spectra were recorded in the range from 50 to 1000 Da at an accumulation time of 0.25 s. MS/MS experiments were conducted using Information-dependent Acquisition-dependent mode (IDA) at an accumulation time of 0.08 s for the 10 most-abundant precursor ions per full scan. Sample acquisition was monitored by the SCIEX OS software Version 2.0.1., released in 2019 (© 2019 AB Sciex). Extraction ion chromatograms (EICs) were generated using the SCIEX OS software. The established parameters were mass accuracy window of 5 ppm, signal-to-noise threshold of 3, minimum area threshold of 1000, minimum intensity threshold of 500.

#### 3.7.1. Screening Workflows

##### Target Screening

A list of 20 target phenolic compounds, namely apigenin, catechin, chlorogenic acid, diosmin, epicatechin, epicatechin gallate, epigallocatechin, gallic acid, hesperidin, isorhamnetin, kaempferol, luteolin, myricetin, myricitrin, naringin, protocatechuic acid, quercetin, quercitrin, rutin, and taxifolin was created. To confirm the identity of the target analytes, the mass accuracies of the precursor ion and the qualifiers, the Rt, and the MS/MS spectra of the real samples and standard solutions were compared.

##### Nontarget Screening

A list of suspect compounds was generated based on the available literature concerning the phytochemical profile of PPs. The mass of the deprotonated ions was calculated based on the molecular formula, and the extracted ion chromatograms were studied using the following parameters: mass accuracy window of 5 ppm; signal-to-noise threshold of 3; minimum area threshold of 1000; minimum intensity threshold of 500. MS/MS fragments were compared to data from SCIEX Natural Products Library for the identification of the unknowns.

### 3.8. GC-MS Analysis

An appropriate amount of the PP extract prepared under the optimum ultrasound-assisted alkaline hydrolysis conditions (0.5 mL) was dried over N_2_ stream in a 1 mL volumetric flask. Immediately after, 100 µL of BSTFA and 900 µL of anhydrous pyridine were added, and the mixture was left to stand for 20 min at room temperature. Then, 1 µL amounts of these solutions were subjected to GC-MS analysis that was carried out using a TraceGC Ultra (ThermoFinnigan, Milan, Italy) gas chromatography coupled with a ISQ (Thermo Fisher Scientific, Milan, Italy) single quadrupole mass spectrometer equipped with a Triplus RSH autosampler supplied by Thermo Fisher Scientific (ThermoFinnigan, Offenbach, Germany). Analytes were separated on a nonpolar HP-5ms silica-fused capillary column coated with 0.25 mm film of poly (dimethylsiloxane) as the stationary phase (Agilent) (HP-5 30 m length × 0.25 mm, film thickness, 0.25 μm). Helium was used as the carrier gas at a constant flow rate of 1.8 mL/min. The oven temperature program used was as follows: initial temperature 40 °C for 2 min, followed by an increase of 10 °C/min for 5 min till 300 °C and then holding the final temperature for 5 min. The total runtime of the GC program was 33 min. The transfer line temperature was kept at 280 °C; EI energy: 70 eV, mass range: 50 to 600 *m*/*z*. The temperature of the MS source was 300 °C. The GC was equipped with a programmable temperature vaporiser injector (PTV) (ThermoFinnigan, Milan, Italy) that was used in the split ratio mode 6:1 at an injection port base temperature of 250 °C. Data acquisition, processing, and evaluation were carried out using the standard software Xcalibur Data System Version 4.2 (ThermoFinnigan, Austin, TX, USA). Compounds were identified by comparison of their retention time and mass spectra with those of available standards or by spectra matching with literature data and NIST library spectra. (NIST Mass Spectral Database, 2011 from National Institute of Standards and Technology, Gaithersburg, MD, USA)

### 3.9. Statistical Analysis

Data were analysed by one-way analysis of variance (ANOVA) using Minitab 15.1.20.0 (Minitab, Inc., State College, PA, USA) software.

## 4. Conclusions

The pomegranate processing industry generates worldwide enormous amounts of by-products, such as pomegranate peels, which constitute a rich source of phenolic compounds. In this view, PP could be exploited as a sustainable source of ellagic acid, which is a compound that possesses various biological actions. Τhe present study aimed at the liberation of ellagic acid from its bound forms via ultrasound-assisted alkaline hydrolysis. The optimum hydrolysis conditions were found to be 32 min, 1:48 v/w, and 1.5 mol/L NaOH. RP-HPLC-DAD, LC-QTOF-MS, and GC-MS analysis of the PP extract obtained under the optimum conditions revealed the presence of various organic acids, sugars, and amino acids, as well as phenolic compounds, e.g., gallic acid, caffeic acid, p-coumaric acid, and ellagic acid. The latter one was liberated from ellagitannins, such as punicalagin, upon hydrolysis. The proposed methodology is expected to add value to the pomegranate processing industry, thereby boosting the local economy, and also could be of use for food, pharmaceutical, and cosmetics applications.

## Figures and Tables

**Table 1:** Experimental design for three-factor five-level CCD and experimental values for the responses of response surface methodology.

	Independent Variables			Dependent Variables *^,^**					
Run	Duration of Sonication (min) (Χ_1_)	Solvent:Solid Ratio (*v*/*w*) (Χ_2_)	Concentration of ΝaOH (mol/L) (X_3_)	TPC (Y_1_) (mg GAE/100 g dry PPs)	A_DPPH_ (Y_2_) (μmol Trolox/100 g PPs)	A_ABTS_ (Y_3_) (μmol Trolox/100 g PPs)	A_CUPRAC_ (Y_4_) (μmol Trolox/100 g PPs)	Punicalagin Content (Y_5_) (mg/100 g PPs)	Ellagic Acid Content (Y_6_) (mg/100 g PPs)
1	90	41	1	3892 ± 37	18,386 ± 803	24,441 ± 1103	37,011 ± 1872	22 ± 2	1426 ± 16
2	25	41	3	4390 ± 17	21,417 ± 533	29,907 ± 1143	26,884 ± 1088	58 ± 5	1560 ± 56
3	58	30	2	1577	9658	23,703	19,230	64	766
4	58	30	2	1562	9653	31,532	17,704	57	794
5	58	30	0.5	3640 ± 94	16,116 ± 651	17,634 ± 283	26,104 ± 1493	47	1803
6	25	18	3	2909 ± 64	11,310 ± 347	13,618 ± 577	15,118 ± 601	62 ± 1	885 ± 20
7	58	10	2	2716 ± 91	16,016 ± 643	18,819 ± 268	31,602 ± 840	72 ± 5	456 ± 17
8	25	18	1	2732 ± 50	69,832 ± 390	15,051 ± 1065	14,566 ± 729	41 ± 6	936 ± 55
9	90	18	1	3114 ± 41	11,163 ± 558	17,626 ± 1004	42,767 ± 1830	34 ± 4	930 ± 34
10	58	30	2	1364	8810	24,453	16,331	45	752
11	90	18	3	3757 ± 209	15,592 ± 630	21,064 ± 1070	28,818 ± 534	56 ± 5	1179 ± 53
12	3	30	2	2530 ± 116	12,837 ± 542	16,165 ± 381	25,688 ± 2250	58 ± 4	674 ± 54
13	58	30	2	1611	9136	26,189	15,225	68	791
14	25	41	1	5114 ± 253	22,211 ± 901	37,272 ± 920	32,168 ± 1080	52 ± 4	1553 ± 64
15	58	30	2	1316	9877	28,079	26,285	59	762
16	90	41	3	4282 ± 260	33,642 ± 777	32,340 ± 1372	31,396 ± 2643	32 ± 4	1828 ± 39
17	112	30	2	3032 ± 146	15,766 ± 191	21,045 ± 627	56,521 ± 220	28 ± 1	834 ± 50
18	58	30	2	1751	9939	29,993	28,841	61	727
19	58	30	4	5976 ± 173	18,709 ± 970	25,541 ± 922	26,501 ± 1078	109 ± 5	228 ± 17
20	58	49	2	4328 ± 103	29,547 ± 769	33,274 ± 1315	29,400 ± 636	48 ± 1	1412 ± 49

* PPs: Pomegranate peels, ** All responses are expressed as mean ± s.d. values (*n* = 3), except for the center points 3, 4, 10, 13, 15, 18, which were measured once.

**Table 2:** Analysis of variance of TPC, A_DPPH_, A_ABTS_, A_CUPRAC_, punicalagin, and ellagic acid content values.

	TPC	A_DPPH_^●^	A_ABTS_^●+^	A_CUPRAC_	Punicalagin Content	Ellagic Acid Content
R^2^ (%)	97.60%	78.98%	85.77%	86.00%	90.50%	99.58%
R^2^adj (%)	95.20%	57.90%	71.54%	72.01%	80.99%	99.17%
R^2^pred (%)	79.99%	0.00%	71.09%	71.99%	79.59%	96.66%
	*p*-value/F-value/DF *					
Regression	0.000/40.68/9	0.051/3.76/9	0.007/6.03/9	0.006/6.14/9	0.001/9.52/9	0.000/239.39/9
Lack of fit	0.067/4.44/4	0.000/969.37/4	0.247/1.91/4	0.500/0.96/4	0.148/5.85/4	0.088/3.80/4
Linear Coefficients	28.13/3	10.26/3	1.66/3	0.29/3	9.44/3	310.08/3
Χ_1_	0.456	0.260	0.578	0.001	0.000	0.001
Χ_2_	0.000	0.765	0.000	0.303	0.014	0.000
Χ_3_	0.210	0.334	0.477	0.089	0.021	0.000
Quadratic Coefficients	92.36/3	3.04/3	3.15/3	7.45/3	2.84/3	401.76/3
X_1_^2^	0.000	0.220	0.030	0.001	0.001	0.668
Χ_2_^2^	0.000	0.033	0.940	0.074	0.320	0.000
Χ_3_^2^	0.000	0.292	0.177	0.597	0.071	0.000
Interactive Coefficients	6.50/3	7.26/3	2.55/3	1.73/3	2.75/3	16.23/3
X_1_X_2_	0.007	0.040	0.081	0.069	0.035	0.198
Χ_1_X_3_	0.059	0.014	0.085	0.371	0.832	0.000
X_2_X_3_	0.148	0.028	0.891	0.877	0.159	0.080

* DF = Degree of Freedom.

**Table 3:** Polynomial equations for TPC, A_ABTS_, A_CUPRAC,_ punicalagin and ellagic acid content responses.

Model	Response	Actual Value of Factors
A	TPC	TPC = 8944.20 − 39.73X_1_ − 209.85X_2_ − 3997.66X_3_ + 0.47X_1_^2^ + 5.70X_2_^2^ + 1010.23X_3_^2^ − 0.86X_1_X_2_ + 6.4X_1_X_3_ − 13.2X_2_X_3_ (Equation (1))
B	A_ABTS_	A_ABTS_ = −10,047.7 + 330.03X_1_ + 907.86X_2_ + 5191.61X_3_ − 2.42X_1_^2^ + 0.58X_2_^2^ − 2054.08X_3_^2^ − 6.83X_1_X_2_ + 81.52X_1_X_3_ − 16.83X_2_X_3_ (Equation (2))
C	A_CUPRAC_	A_CUPRAC_ = 23,319.2 − 62.99X_1_ − 647.22X_2_ + 3658.91X_3_ + 6.43X_1_^2^ + 23.01X_2_^2^ − 1167.55X_3_^2^ − 10.89X_1_X_2_ − 60.04X_1_X_3_ + 28.59X_2_X_3_ (Equation (3))
D	Punicalagin Content	Punicalagin Content = −18.5 + 0.96X_1_ + 1.76X_2_ + 33.2709X_3_ − 0.01X_1_^2^ − 0.01X_2_^2^ − 4.72X_3_^2^ − 0.01X_1_X_2_ + 0.02X_1_X_3_ − 0.3X_2_X_3_ (Equation (4))
E	Ellagic Acid Content	Ellagic Acid Content = 2607.45 − 3.24X_1_ − 6.71X_2_ − 2115.82X_3_ + 0.004X_1_^2^ + 0.51X_2_^2^ + 481.16X_3_^2^ − 0.05X_1_X_2_ + 2.82X_1_X_3_ +2.4X_2_X_3_ (Equation (5))

**Table 4:** Optimum values of the duration of ultrasound-assisted alkaline hydrolysis, solvent:solid ratio, and NaOH concentration, as well as predicted and experimental response values.

Factor	Optimum Actual Values	Predicted Values	Mean Experimental Values *
Duration (min)	32	TPC (mg GAE/100 g dry PPs)	
		4987	4230 ± 190
		A_ABTS_ (μmol Trolox/100 g dry PPs)	
Solvent: Solid Ratio (*v*/*w*)	48:1	36,230	32,398 ± 1817
		A_CUPRAC_ (μmol Trolox/100 g dry PPs)	
		31,860	29,816 ± 1955
NaOH Concentration (mol/L)	1.5	Punicalagin Content (mg/100 g dry PPs)	
		56	59 ± 3
		Ellagic Acid Content (mg/100 g dry PPs)	
		1477	1457 ± 71

* All responses are expressed as mean ± s.d. values (*n* = 3).

**Table 5:** Identified phenolic compounds in the PP extract prepared under the optimum hydrolysis conditions with the aid of LC-QTOF-MS.

Retention Time (min)	Adduct Type	Chemical Formula	Concentration (μg/mL)	Compound Name
Target analytes				
4.92	[M − H]^−^	C_15_H_14_O_6_	0.09	Catechin
7.07	[M − H]^−^	C_27_H_30_O_16_	0.22	Rutin
8.49	[M − H]^−^	C_15_H_10_O_7_	0.05	Quercetin
8.77	[M − H]^−^	C_15_H_10_O_6_	0.11	Luteolin
9.33	[M − H]^−^	C_15_H_10_O_6_	0.13	Kaempherol
9.45	[M − H]^−^	C_15_H_10_O_5_	0.29	Apigenin
6.46	[M − H]^−^	C_15_H_12_O_7_	0.05	Taxifolin
4.11	[M − H]^−^	C_7_H_6_O_4_	1.04	Protocatechuic acid
Non-target analytes				
4.89	[M − H]^−^	C_9_H_8_O_4_	Not quantified	Caffeic acid
6.49	[M − H]^−^	C_10_H_10_O_4_	Not quantified	Ferulic acid
7.34	[M − H]^−^	C_14_H_6_O_8_	Not quantified	Ellagic acid
7.51	[M + H]^−^	C_9_H_8_O_3_	Not quantified	*p*-Coumaric acid

**Table 6:** Compounds identified by GC-MS analysis in the PP extracts prepared without or with ultrasound-assisted alkaline hydrolysis.

Compounds	Retention Time (Rt)	*m*/*z*
Amino acids		
Glycine	8.37	174,145,130
Cystathionine	9.65	366,206,147
Organic acids		
Succinic acid	12.09	147,247,73
Fumaric acid	12.59	245,147,75
Malic acid	14.37	147,233,245
Citric acid	18.27	273,147,363
Sugar alcohols		
Myo-inositol	19.87	305,217,147
Saccharides (mono- and di-)		
Fructose	18.19	437,217,204
D-Glucose	18.93	204,191,217
D-Sorbitol	19.30	319,205,147
D-Mannitol	19.34	319,205,147
Phenolic acids		
Vanillic acid	17.58	297,312,267
p-Coumaric acid	17.86	308,293,219
Isoferulic acid	19.20	338,323,308
Gallic acid	19.58	458,281,73
Caffeic acid	21.32	396,381,219
Sinapinic acid	22.26	368,338,353
Ellagic acid	30.19	575,487,73

**Table 7:** Levels of independent variables in coded and uncoded values used in the experimental design.

Symbols	Variable			Level		
				Coded value		
		−a	−1	0	+1	+a
				Uncoded value		
Χ_1_	Duration of sonication (min)	3	25	58	90	112
Χ_2_	Solvent:solid ratio (*v*/*w*)	10:1	18:1	30:1	41:1	49:1
Χ_3_	Concentration of NaOH (mol/L)	0.5	1	2	3	4

## Data Availability

The data presented in this study are available on request from the corresponding author.

## Supplementary Materials

The following supporting information can be downloaded at: https://www.mdpi.com/article/10.3390/molecules29112424/s1, Table S1. Chromatograms and MS/MS fragmentation patterns of the target and non-target analytes.
