# Supplementary material for: Recovery of Ellagic Acid from Pomegranate Peels with the Aid of Ultrasound-Assisted Alkaline Hydrolysis
Source: Molecules. 2024 May 21;29(11):2424. doi: 10.3390/molecules29112424 (PMC11173712; doi:10.3390/molecules29112424)

Table S1. Chromatograms and MS/MS fragmentation patterns of the target and non-target analytes.

	Chromatograms	MS/MS Fragmentation Patterns
	**Target analytes**	
**Apigenin**	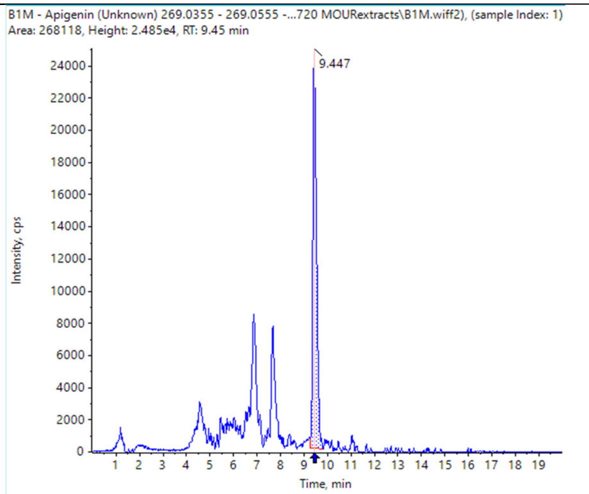	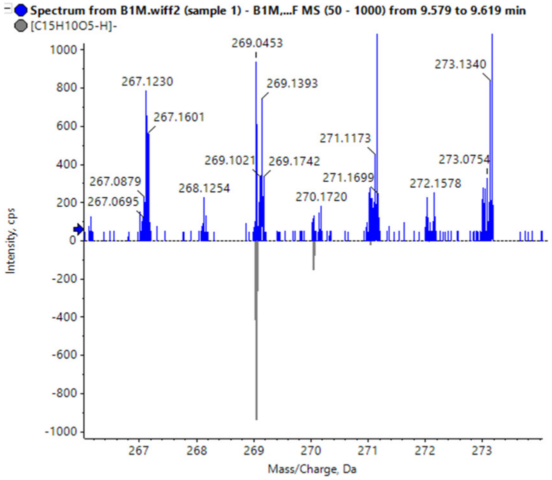
**Catechin**	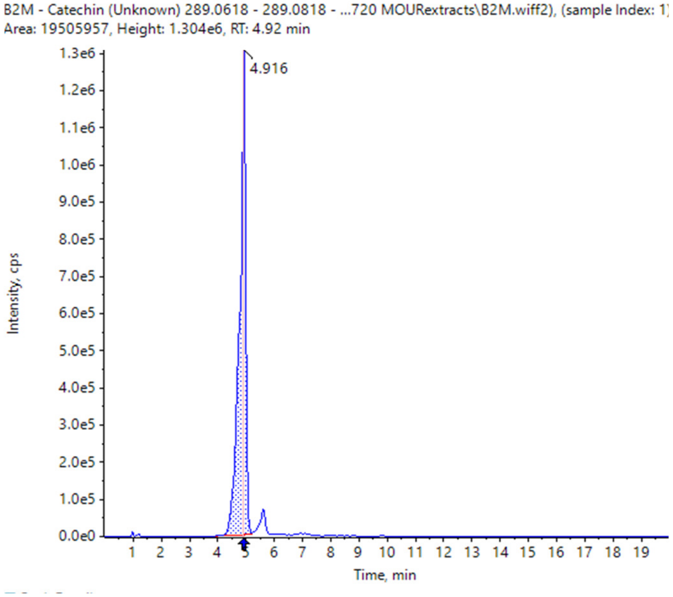	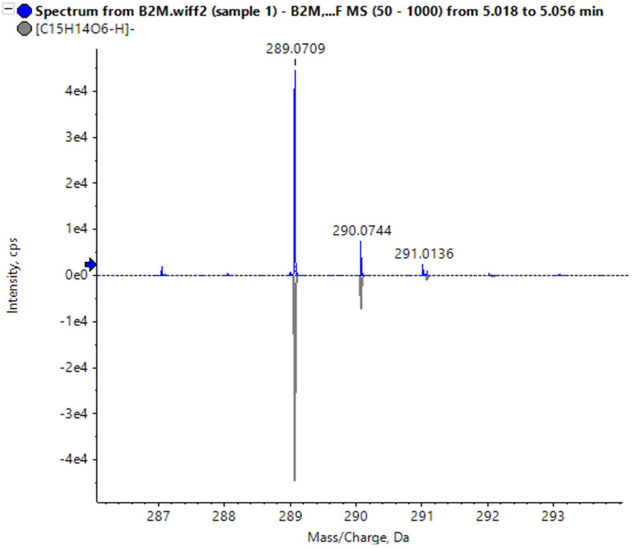

Kaempferol

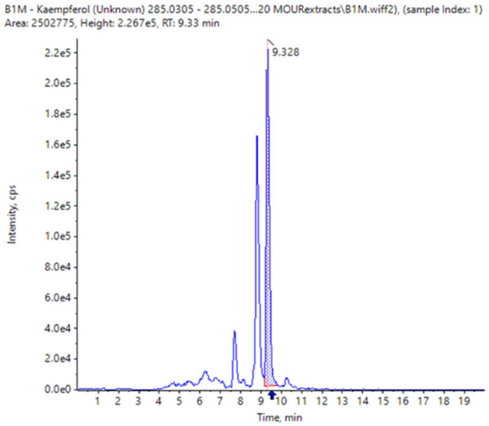

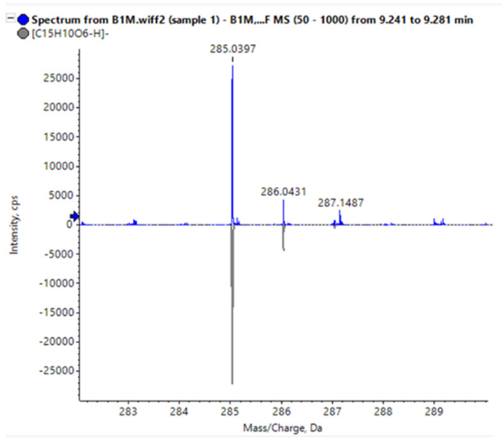

Luteolin

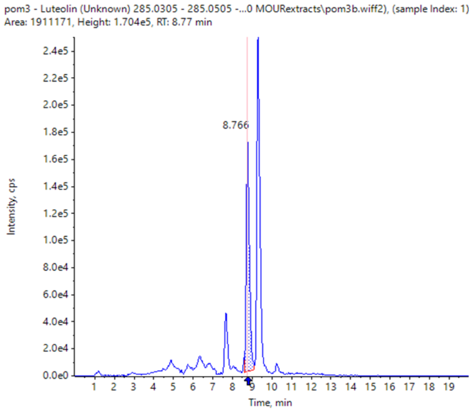

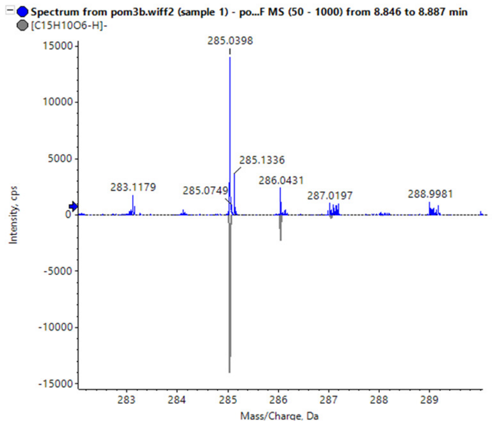

Protocatechuic acid

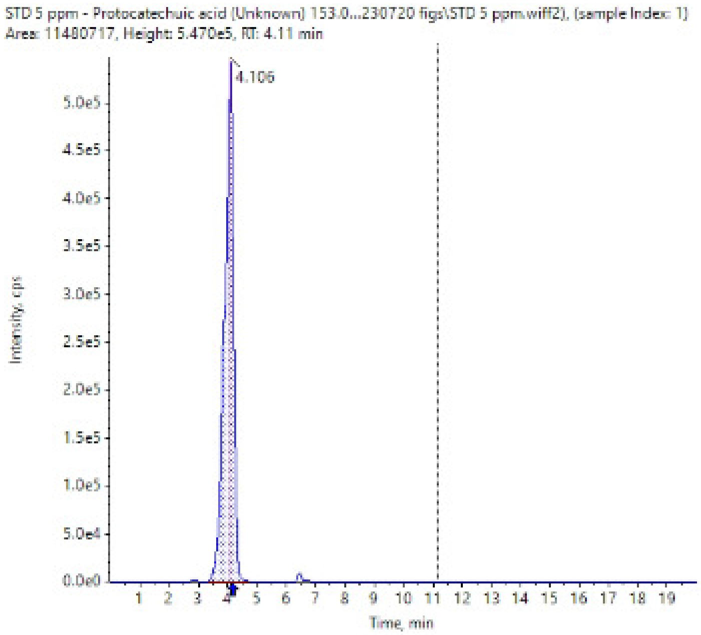

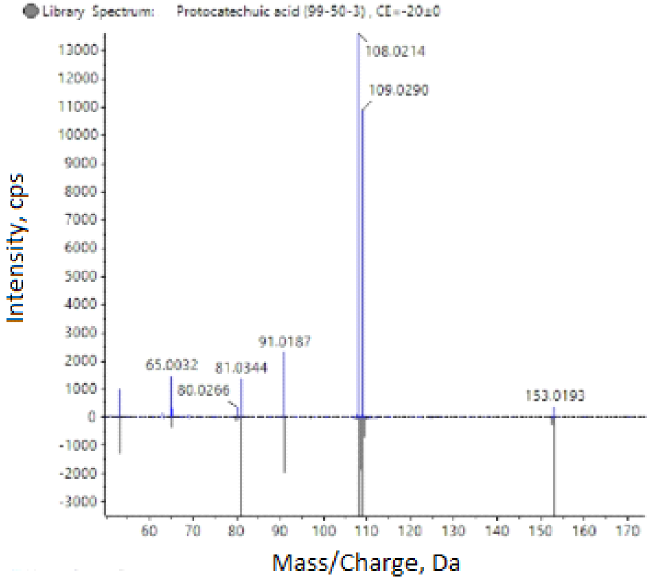

Quercetin

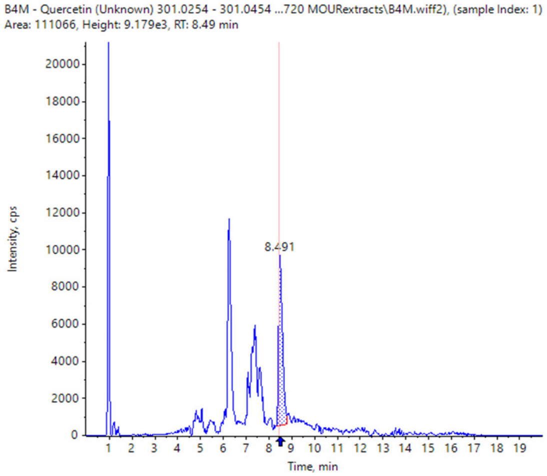

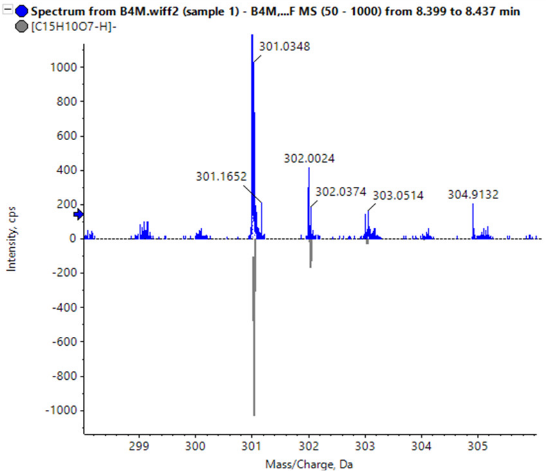

Rutin

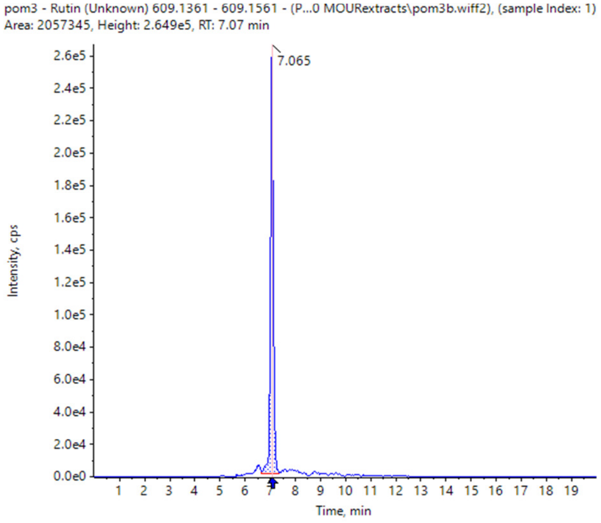

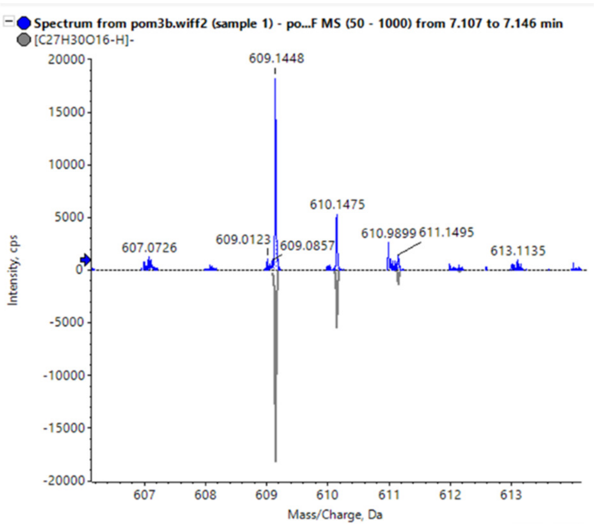

Taxifolin

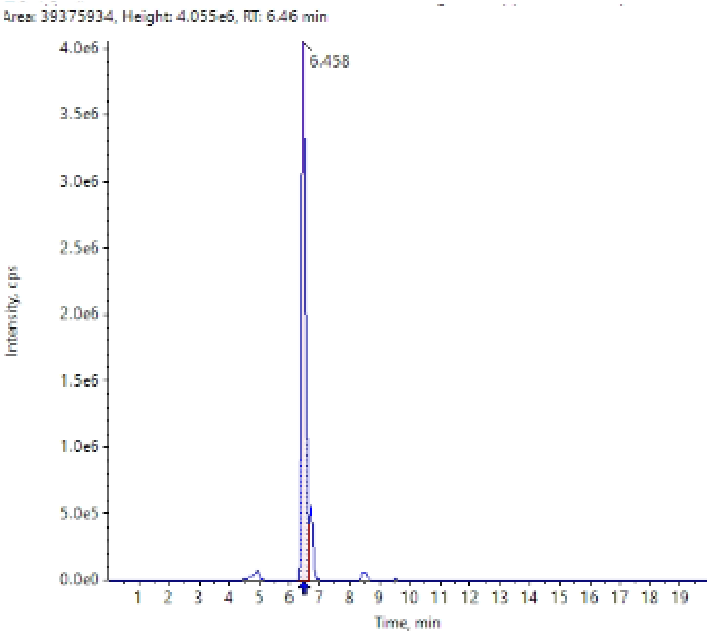

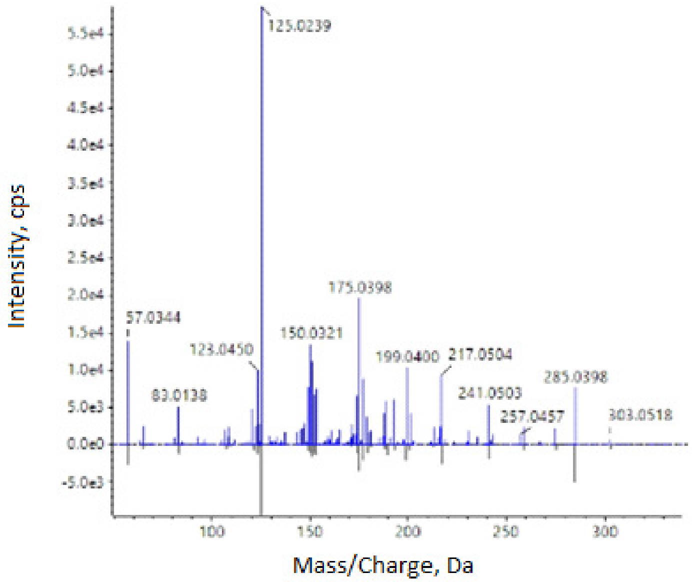

Tentatively identified

Ellagic acid

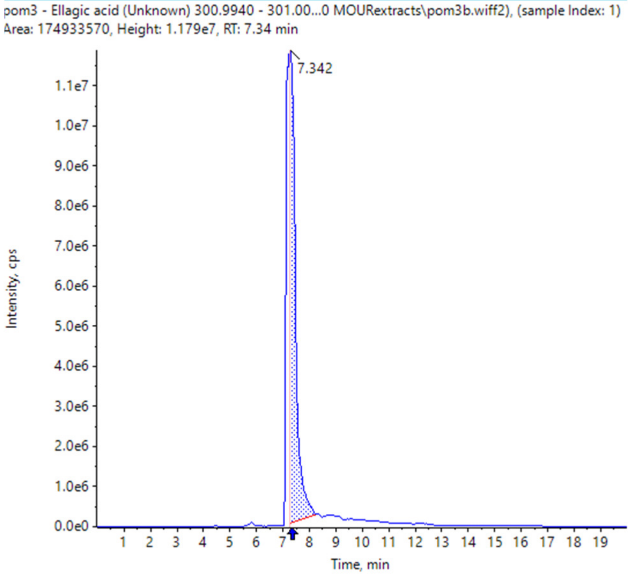

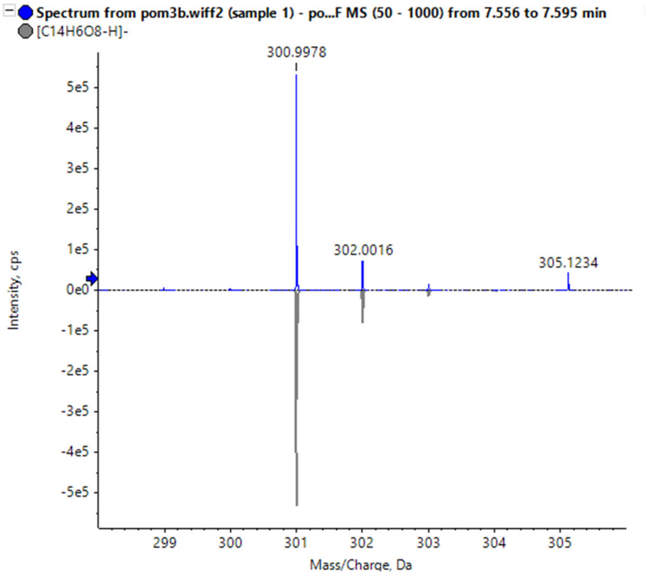

Ferulic acid

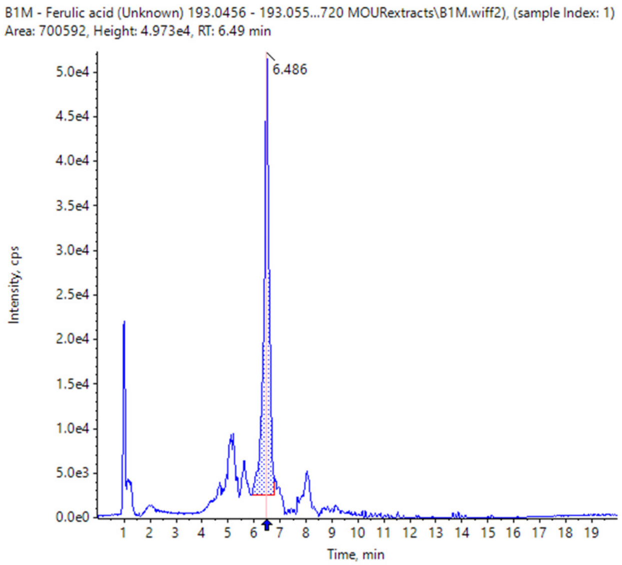

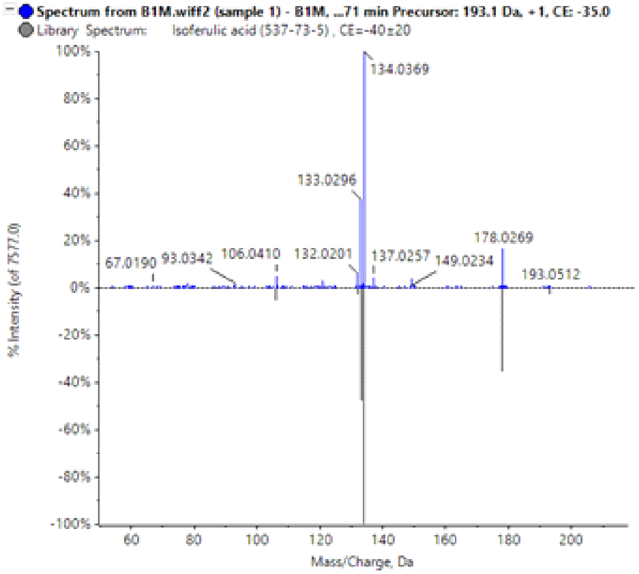

Caffeic acid

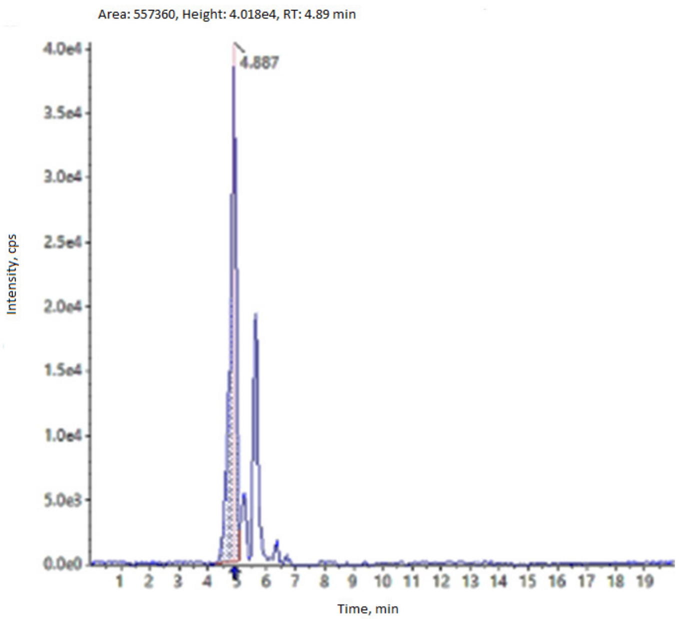

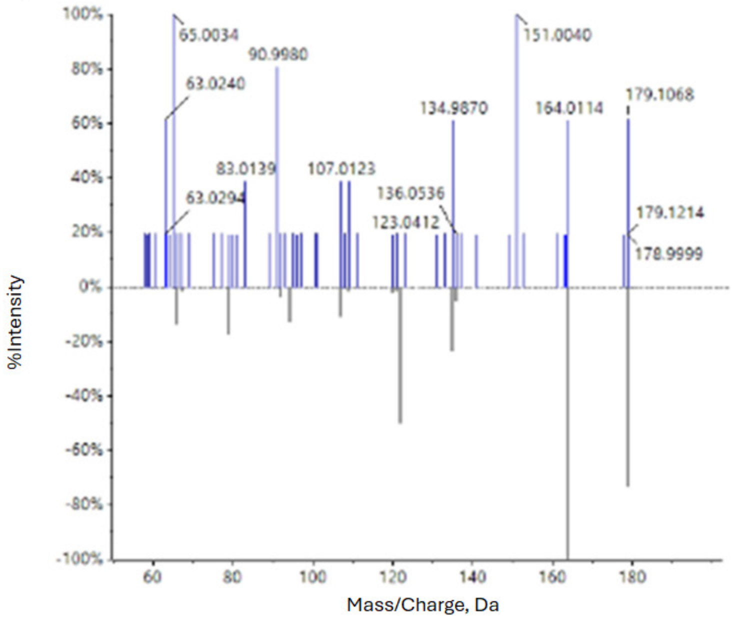

***p*-Coumaric acid**

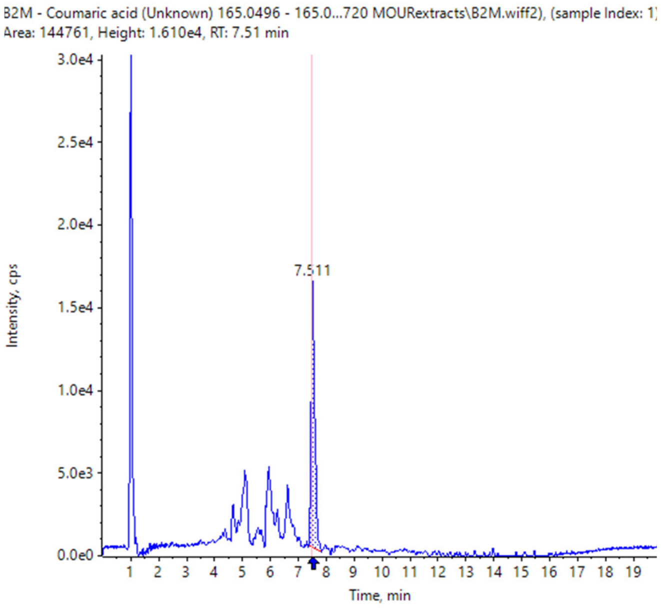

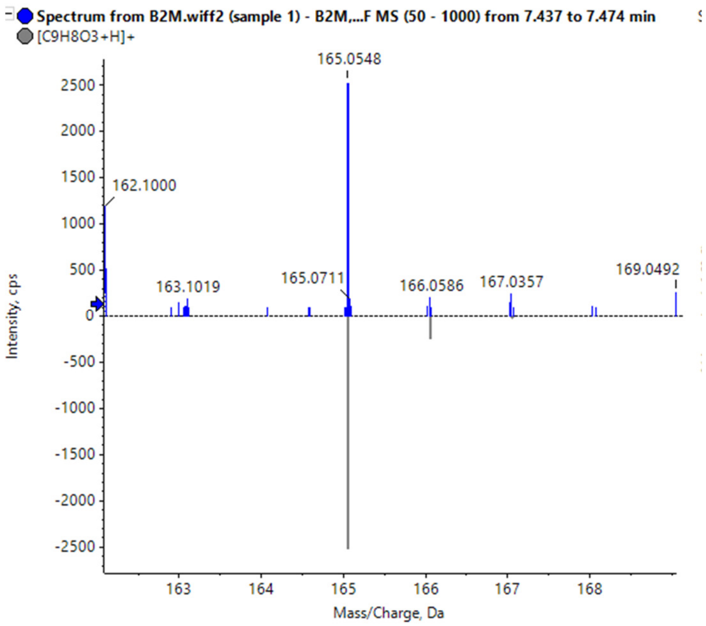

Supplement: Supplementary file 1 [file molecules-29-02424-s001.zip › molecules-2964366-supplementary.pdf]
